# Energy-Dispersive X-Ray Diffraction: Principles, Instrumentation and Emerging Applications

**DOI:** 10.3390/ma19040697

**Published:** 2026-02-12

**Authors:** Zhimao Wang, Gang Li, Jie Zhang, Yanping Wang, Rui Sun, Jiayang Lin

**Affiliations:** Institute of High Energy Physics, Chinese Academy of Sciences, Beijing 100049, China

**Keywords:** energy-dispersive X-ray diffraction (EDXRD), materials characterization, synchrotron radiation, detectors technology, operando spectroscopy

## Abstract

Energy-Dispersive X-ray Diffraction (EDXRD) employs a polychromatic (white) X-ray beam and an energy-discriminating detector at a fixed scattering geometry to measure diffracted intensity as a function of photon energy. This technique enables the rapid acquisition of diffraction data over a wide range of d-spacings without mechanical scanning of the scattering angle, making it particularly valuable for time-resolved, bulk-penetrating, and operando studies. In this review, we provide a comprehensive overview of EDXRD, covering the fundamental principles and underlying physics, experimental methodologies and data analysis workflows, synchrotron white-beam implementations compared to monochromatic approaches, detector strategies, parameter optimization for accurate and efficient measurements, and representative applications in high-pressure science and battery research. Finally, we discuss current challenges and future prospects, including advances in detector technology, machine learning-assisted spectral analysis, and the development of standardized, automated EDXRD systems.

## 1. Introduction

X-ray diffraction (XRD) remains one of the most fundamental and widely applied techniques in the structural sciences. Since the discovery of X-ray scattering by crystals, diffraction methods have provided unparalleled insights into atomic arrangements, lattice symmetry, and the evolution of matter under external stimuli [1,2,3,4,5]. Traditionally, XRD has been implemented using the angle-dispersive (ADXRD) approach, where a monochromatic incident beam is scattered by a sample and diffracted intensities are recorded as a function of scattering angle [6,7,8,9,10,11]. While ADXRD is powerful, it also presents limitations, including the need for angular scanning, relatively long acquisition times, and restricted penetration when dealing with highly absorbing or bulky environments [12,13,14,15].

To overcome these constraints, the energy-dispersive approach (EDXRD) was introduced [16,17,18]. In EDXRD, a polychromatic (“white”) incident beam is employed, and diffracted photons are analyzed according to their energy rather than angle [19,20,21]. By fixing the scattering geometry and dispersing the energy spectrum, EDXRD yields complete diffraction patterns without mechanical scanning [16]. This configuration enables rapid acquisition, making the technique uniquely suited for in situ and operando investigations of structural dynamics.

The utility of EDXRD has expanded dramatically with the rise of high-flux, high-brilliance synchrotron radiation sources [22,23,24,25,26]. Modern storage rings, insertion devices, and advanced beamline optics provide intense white beams spanning broad energy ranges (20–200 keV) [22], ideally matched to energy-dispersive measurements. In parallel, detector technology has advanced from early high-purity germanium detectors to silicon drift detectors, cadmium telluride arrays, and pixelated hybrid photon-counting devices [22,27,28]. Together, these innovations now enable EDXRD to probe materials under extreme conditions—such as megabar pressures in diamond anvil cells—while also supporting the real-time monitoring of industrial processes [24,25].

This review provides a comprehensive and critical assessment of EDXRD as a modern scientific tool. We begin with its theoretical foundations, then examine instrumentation and experimental methodologies, with emphasis on synchrotron-based implementations. Particular attention is devoted to detector selection, performance trade-offs, and strategies for optimizing measurement parameters. Representative applications across physics, chemistry, materials science, and engineering are highlighted, followed by a perspective on future directions, including the impact of diffraction-limited, the development of high-speed pixelated detector arrays, and the integration of advanced data analytics and machine learning into diffraction workflows.

## 2. Fundamental Principles

The foundation of EDXRD lies in the reformulation of Bragg’s law in terms of photon energy [4]. The conventional expression:

(1) nλ=2dsinθ

combined with the relation between photon wavelength and energy (*λ* = *hc*/*E*) [29,30], yields:

(2) $${d\prime }_{hkl}=\frac{nhc}{2Esin\theta \;}$$

This expression explicitly relates the lattice plane spacing *d’_hkl_* to the photon energy E detected at a fixed diffraction angle *θ*. *n* is an integer and is referred to as the order of the diffraction, with *n* = 1 being the first order, and *n* = 2 being the second order, and so on [31]. In practical applications, this means that under a fixed diffraction angle *θ*, variations in photon energy can be directly mapped to different d spacings without requiring angle scanning. Consequently, a single detector positioned at a fixed angle can simultaneously record diffraction signals from multiple lattice planes, with the entire diffraction spectrum encoded along the photon energy axis.

In crystallographic convention, an n-order reflection from planes with spacing *d’_hkl_* is strictly equivalent to a first-order reflection from planes indexed as (*nh*, *nk*, *nl*) with spacing *d’_hkl_*/*n*. For example, the second-order diffraction from (100) corresponds exactly to the first-order reflection from (200) [3,32,33]. Consequently, higher-order reflections do not provide independent structural information but are simply reindexed as first-order reflections from planes of reduced spacing. This equivalence underpins the standard practice in X-ray diffraction analysis of considering only first-order reflections.

With: $d_{hkl}=\frac{{d^{\prime }}_{hkl}}{n}$

Bragg’s law may be written in the form:

(3) $$d_{hkl}=\frac{hc}{2Esin\theta \;}$$

This form will be used throughout this article. Overall, Bragg’s law expressed in energy space provides the conceptual basis for EDXRD: it transforms a traditionally angularly scanned measurement into a spectroscopic one, enabling the simultaneous acquisition of diffraction information at a fixed geometry.

## 3. Experimental Methodologies

EDXRD relies on specialized experimental strategies to fully exploit its capability for rapid and in situ structural characterization. This section summarizes the essential methodologies, including instrumentation, sample preparation, data acquisition, data processing, and parameter optimization.

### 3.1. Instrumentation Setup

A typical EDXRD configuration employs a polychromatic (“white”) X-ray source in combination with a fixed scattering angle. The diffracted intensity is recorded by an energy-sensitive detector, such as a high-purity germanium (HPGe) or silicon drift detector (SDD), which provides high energy resolution over a broad spectral range [27,28,34,35,36]. This arrangement enables the simultaneous collection of multiple diffraction peaks across a wide d-spacing range. Depending on the photon source, EDXRD experiments are generally classified into laboratory-based and synchrotron-based setups.

**(a)**  **Laboratory-based configurations**

Laboratory EDXRD systems typically use conventional X-ray tubes with tungsten, molybdenum, or silver anodes as broadband sources [37,38]. These instruments are compact and accessible, making them suitable for routine studies and preliminary structural assessments. However, their usable energy range is restricted by both source output and detector efficiency, often limiting penetration depth and temporal resolution [39]. To improve spectral purity and reduce background, beam conditioning optics such as collimators and filters are applied. Laboratory setups are commonly configured for bulk material studies, biological tissues, or metal material research [39,40,41].

**(b)**  **Synchrotron-based configurations**

At synchrotron radiation facilities, bending magnets, wigglers, or undulators provide highly brilliant white X-ray beams spanning tens to hundreds of keV [42,43,44]. The broad spectral coverage and intense flux significantly enhance the signal-to-noise ratio and allow millisecond-scale data acquisition, which is essential for operando and time-resolved studies [45]. Synchrotron-based EDXRD setups are widely integrated with specialized sample environments, including diamond anvil cells, high-temperature, enabling experiments under extreme conditions [46,47].

**(c)**  **Detector placement and downstream configuration**

**Figure 1:**
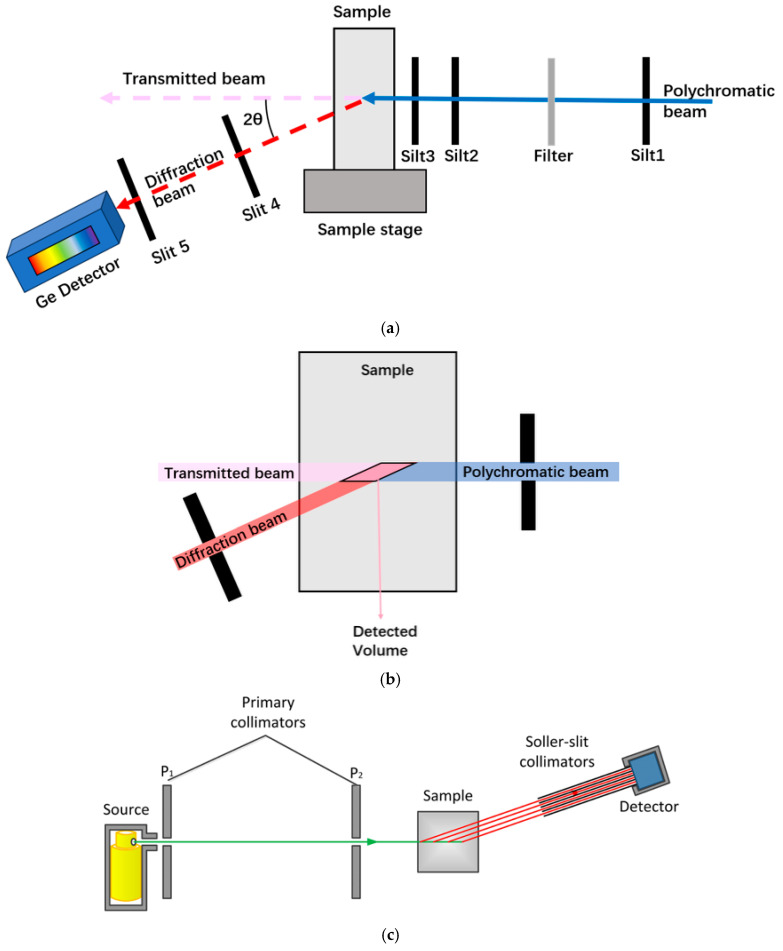
(**a**) Schematic of the EDXRD experimental setup. (**b**) Schematic showing the EDXRD detected volume. (**c**) Schematic showing X-ray tube experimental setup of the EDXRD system [48].

In both laboratory and synchrotron settings, the downstream arrangement is critical for optimizing performance. Collimators and slits are used to define the gauge volume and suppress scattering from outside the sample region, as shown in Figure 1. Energy-sensitive detectors are positioned at a fixed scattering angle, though the choice depends on the desired d-spacing resolution and energy coverage. For high-energy studies (>100 keV), thick germanium detectors are preferred due to their superior quantum efficiency [35], while silicon detectors offer advantages at lower energies [34]. The downstream setup must therefore balance resolution, flux, and spatial selectivity according to experimental objectives.

### 3.2. Sample Preparation

Sample preparation strategies depend on the material form and experimental environment. Powder samples are typically pressed into pellet form or encapsulated in thin-walled capillary tubes to maintain their shape [49]. Bulk materials can be studied directly if the geometry permits [50,51]. For high-pressure research, diamond anvil cells (DACs) are widely employed, while furnaces, cryostats, or environmental chambers are used for temperature or atmosphere-dependent studies [52,53,54]. Careful optimization of sample thickness and homogeneity is essential to balance absorption losses with sufficient diffraction signal.

### 3.3. Data Acquisition

During measurement, the incident beam is collimated and aligned to minimize background contributions. The scattering angle is fixed, typically in the range of 2θ = 5–20° [55], although the choice depends on the d-spacing range of interest. Energy-dispersive spectra are collected over a broad energy interval, and counting times are tailored to the scattering strength of the specimen and the required temporal resolution. For static studies, longer exposures improve signal-to-noise ratios, whereas time-resolved and operando experiments benefit from shorter collection times. However, acquisition time is constrained by the signal-to-noise ratio and detector processing time, making it difficult to increase. Currently, it can reach 10 s or even shorter [45,55].

### 3.4. Data Processing and Analysis

**Figure 2:**
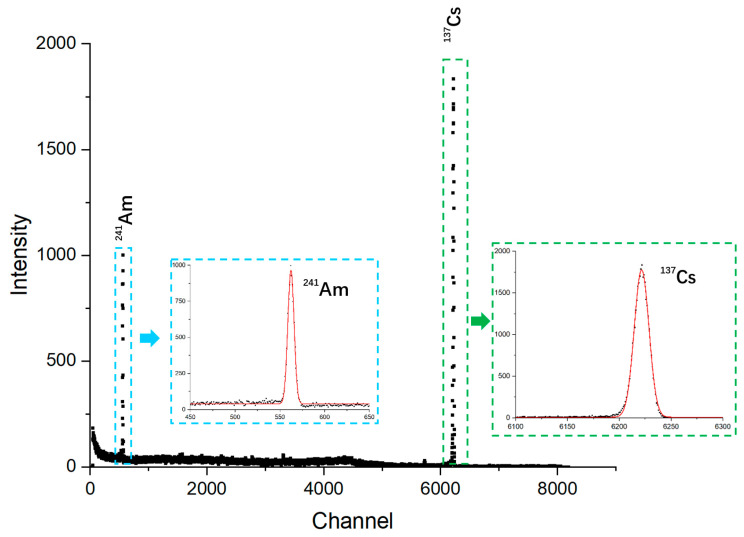
Calibration of a HPGe detector using radionuclide source ^137^Cs and ^241^Am.

The raw energy-dispersive spectra must undergo multiple corrections prior to structural analysis, including geometrical corrections, self-absorption correction, true coincidence summing correction, and compensation for detector efficiency variations [56,57,58,59,60]. Energy calibration is typically performed using radioactive γ- or X-ray sources with well-known emission energies (e.g., ^60^Co, ^133^Ba, ^137^Cs ^226^Ra, ^232^Th, ^241^Am) by measuring the corresponding peak positions [57,58]. Figure 2 illustrates the calibration process of an 8192-channel HPGe detector using radionuclide source ^137^Cs and ^241^Am. The approximate channel number of ^241^Am is 562.4, corresponding to an energy of 59.54 keV. The approximate channel number of ^137^Cs is 6221.6, corresponding to an energy of 661.62 keV. A linear fit between the peak positions and the known energies yields the channel–energy relationship, enabling the conversion of channel numbers into absolute energy values (eV or keV). In this case, it is 106.39 eV per channel. After calibration, structural refinement is carried out depending on whether the target information concerns long-range or local order, employing methods such as Rietveld refinement [41], profile fitting [50,51], etc.

Rietveld refinement is a whole-pattern fitting approach in which structural, instrumental, and sample-related parameters are simultaneously optimized by minimizing the difference between the calculated and experimental powder diffraction patterns. In energy-dispersive X-ray diffraction (EDXRD), diffraction data are collected as a function of photon energy at a fixed scattering angle. The energy dispersion of the incident and detected X-ray beam is incorporated through an instrumental resolution function that accounts for detector energy resolution and beam energy spread, which is calibrated using standard reference materials.

X-ray absorption is treated as an energy-dependent effect and corrected using tabulated mass attenuation coefficients, considering the sample composition and effective path length under the fixed geometry. Fluorescence mainly contributes to a smooth, energy-dependent background in EDXRD, particularly when the incident energy exceeds the elemental absorption edges and is modeled using polynomial or empirical background functions without interfering with Bragg peak profiles.

**Figure 3:**
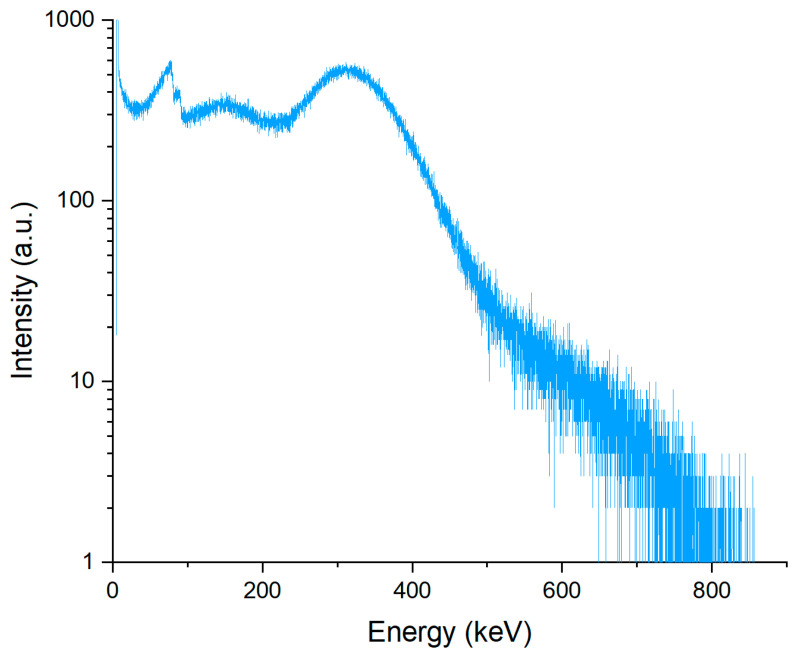
Scattering spectrum measured in air at High Energy Photon Source (HEPS).

After establishing the channel–energy relationship, an air-scattering spectrum was measured at the HEPS test beamline with a 15 mm thick lead slab placed in the beam path. The HPGe detector was positioned at a 45° angle relative to the beam direction and protected by lead sheets. The incident beam from HEPS produced significant scattered photons: measurable photon flux persists well above 300 keV (Figure 3), confirming that the source provides X-rays up to and beyond 300 keV. In the 70–90 keV region, a broad and structured feature was observed. Its origin can be attributed to a combination of Pb characteristic radiation and absorption-edge effects (Pb Kα ≈ 74.96 keV, Pb Kβ ≈ 84.94 keV, Pb K-edge ≈ 88.0 keV) [61], convolved with detector response, partial re-absorption of fluorescence in the lead slab, and scattering/fluorescence contributions from other materials in the experimental hutch. Therefore, the observed structure reflects both photoelectric processes in the lead slab and the complex experimental environment rather than a single, isolated emission line.

## 4. Synchrotron Implementations

Synchrotron radiation provides a continuous spectrum determined by the bending magnet or undulator/wiggler source. White beams, which do not employ a monochromator, deliver a broad, high-flux photon output spanning from a few keV up to several hundred keV [42,43,44]. In contrast, monochromatic beams utilize crystal optics to select a narrow energy band, forming the basis of ADXRD experiments [62,63,64], which typically require fine angular diffraction scans to sample.

### 4.1. Advantages of Synchrotron White Beam for EDXRD

(a) Photon Flux

White beams deliver a substantially higher integrated photon flux than monochromatic beams, since no energy selection is imposed by a monochromator. In contrast, monochromatization typically reduces the incident photon flux by approximately five to six orders of magnitude [65,66]. The exceptionally high flux of white beams enables rapid data acquisition and allows complete diffraction spectra to be collected on much shorter timescales, with the ultimate limitation set primarily by detector response and electronics rather than by mechanical motion.

(b) Experimental Geometry

The fixed scattering geometry of white-beam EDXRD eliminates the need for continuous angular scanning, reducing mechanical complexity and facilitating the integration of complex sample environments (e.g., furnaces, gas cells, or diamond anvil cells) [46,47]. This stability is particularly advantageous for in situ and operando studies conducted under extreme or dynamic conditions.

(c) Penetration Depth

The broad energy range of white beams includes high-energy photons (>50–300 keV) with significant penetration capability. This capability enables the investigation of thick, encapsulated, or metallic samples, thereby extending the applicability of EDXRD to engineering materials and devices.

### 4.2. Limitations of Synchrotron White Beam for EDXRD

The primary limitation of white-beam EDXRD lies in the finite energy resolution of the detector. For example, high-purity germanium (HPGe) detectors typically achieve an energy resolution of 0.19 keV at 5.9 keV [67], corresponding to ΔE/E ≈ 3.2 × 10^−2^. In contrast, monochromatic ADXRD can reach ΔE/E on the order of 10^−4^ using Si crystal monochromators [65,68]. This difference translates into broader diffraction peak profiles in EDXRD, reducing the precision of lattice parameter determination and hindering the resolution of subtle features such as peak splitting or small distortions. Consequently, for studies requiring ultimate accuracy in lattice constants and detailed texture analysis, monochromatic ADXRD remains superior.

Furthermore, for ADXRD, the choice between white-beam and monochromatic-beam diffraction also depends on the average grain size of the sample relative to the beam footprint. For fine-grained samples, where the grain size is much smaller than the beam, monochromatic beams are preferred, producing Debye–Scherrer ring patterns from thousands to millions of crystallites. For coarse-grained samples, where the grain size is comparable to or larger than the beam, white-beam Laue diffraction is advantageous, capturing reflections over a broad energy range in a single exposure and providing rich microstructural information without requiring sample or detector rotation [69]. White-beam Laue diffraction, typically performed with imaging plates or area detectors, differs from EDXRD, which relies on energy-dispersive detectors. Nevertheless, both techniques exploit the high photon flux of synchrotron white beams and share the advantages of simplified experimental geometry.

For EDXRD, fine-grained samples similarly benefit from the ability to capture a wide range of reflections in a single exposure, without rotating the sample or detector. However, as noted above, the achievable resolution is ultimately limited by the energy resolution of the detector.

## 5. Detector Strategies

### 5.1. Operational Principles of X-Ray Detectors

EDXRD relies on detectors that convert incident photons into measurable electrical signals. High-purity germanium (HPGe) detectors are the most widely employed for high-resolution applications. In these devices, incident X-rays generate electron–hole pairs within a semiconductor volume, with the number of pairs proportional to the photon energy. This band gap in a germanium crystal is 0.67 eV at 77 K, but an average energy of 2.96 eV is required to create a free e–h pair [70,71]. An applied bias collects the carriers at electrodes, producing pulses whose amplitudes correspond to deposited energy. The primary performance metrics include energy resolution, efficiency, count rate capability, and electronic noise, etc. [72,73,74] HPGe detectors require cooling—traditionally with liquid nitrogen (~77 K)—to suppress leakage current and thermal noise. Recent developments in mechanical cryocoolers and long-life cryo-systems have greatly simplified operation for synchrotron and laboratory beamlines.

### 5.2. Detector Types and Selection Criteria

While HPGe detectors offer superior energy resolution, alternative detectors are often used depending on the experimental requirements:

HPGe: They offer excellent energy resolution at low energy, and maintain high detection efficiency over a broad energy range, extending up to 1.33 MeV. These characteristics make them particularly suitable for resolving complex spectra in energy-dispersive X-ray diffraction and related applications. Although HPGe detectors have suffered from limited count rate capability due to long shaping times and dead-time losses, the development of modern digital pulse processors and segmented detector arrays has significantly mitigated these constraints, enabling operation at higher photon fluxes without severe resolution degradation [67,75].

CdTe/CZT detectors: Cadmium telluride (CdTe), due to its high atomic numbers (Z_Cd_ = 48, Z_Te_ = 52), provides strong photoelectric absorption and thus represents a favorable material for X-ray sensor fabrication. A key advantage of CdTe detectors is their ability to operate at or near room temperature, eliminating the need for cryogenic cooling that is essential for germanium-based detectors. Energy resolution is lower than HPGe but improving, reaching values 258 eV FWHM at 5.9 keV [76,77].

Silicon Drift Detectors: SDDs provide excellent quantum efficiency in the low-energy regime and are well-suited for the efficient detection of X-rays below 30 keV. They combine high energy resolution, reaching values as low as 122.7 eV FWHM at 5.9 keV. When coupled with advanced application-specific integrated circuits (ASICs), SDDs achieve stable performance, enabling their widespread use in synchrotron-based experiments, material science, and microanalysis [34,78,79].

Table 1 summarizes the comparison results of several detectors at energies of 5.9 keV and 122 keV. It can be observed that at 5.9 keV, the SDD detector exhibited the best energy resolution, followed by the HPGe detector, while some CZT detectors demonstrated performance comparable to that of the HPGe detector. At the higher energy of 122 keV, the HPGe detector showed superior energy resolution compared with the CZT detector.

**Figure 4:**
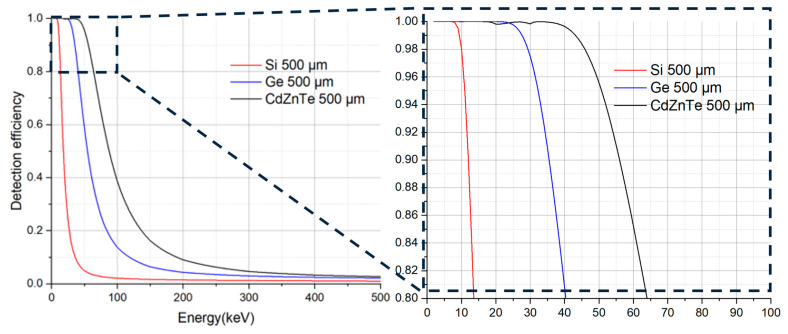
Calculated detection efficiencies as a function of photon energy.

The influence of the atomic number on X-ray absorption efficiency is clearly illustrated in Figure 4, where the detection efficiency as a function of photon energy is compared across different sensor materials, assuming a uniform sensor thickness of 500 µm. For photon (X-ray/γ-ray) detectors, their intrinsic detection efficiency is typically approximated as the absorption efficiency of photons at different energies for a given material thickness [80]. In general, materials with higher atomic numbers exhibit superior stopping power and thus achieve higher detection efficiency, particularly at moderate to high photon energies. Cadmium zinc telluride (CdZnTe) demonstrated the highest efficiency among the compared materials; however, absorption edges must be considered when evaluating performance across different energy regions. For example, near the Te K edge (~31 keV), the efficiency of CdZnTe decreases. This is attributed to the proportions of the three elements, particularly the ratio between Cd and Zn. This underscores the importance of matching detector material properties not only to the overall energy range of interest and energy resolution but also to specific spectral regions where absorption edge effects may dominate.

**Table 1:** Comparison of energy resolution among different detectors.

Detector	Resolution (eV)	
	@5.9 keV FWHM	@122 keV FWHM
SDDs + SIRIO [79]	122.7	--
SDDs + RIGEL [81]	167	--
SDDs + SCARLET [82]	171.5	--
HPGe GLP type [67]	190	510
HPGe [83]	400	680
CZT [77]	258	1770
CZT [84]	311	--
CdTe [85]	--	6200

## 6. Parameter Optimization

### 6.1. Beam Conditioning and Beamline Configuration

Synchrotron white beams must be carefully conditioned to match the experimental requirements. A typical EDXRD beamline layout includes the radiation source (bending magnet, wiggler, or undulator), transport optics with optional filters, precision slits/collimators, a sample stage with environmental control (temperature, gas flow, or pressure cell), the detector system (commonly HPGe or similar alternatives), and data acquisition electronics.

Gap: For a synchrotron wiggler source, a smaller gap corresponds to a stronger magnetic field, leading to a higher deflection parameter (*K*). As *K* increases, the electron beam experiences stronger oscillations, resulting in both higher photon flux and an increased upper limit of the photon energy spectrum [86].

**Figure 5:**
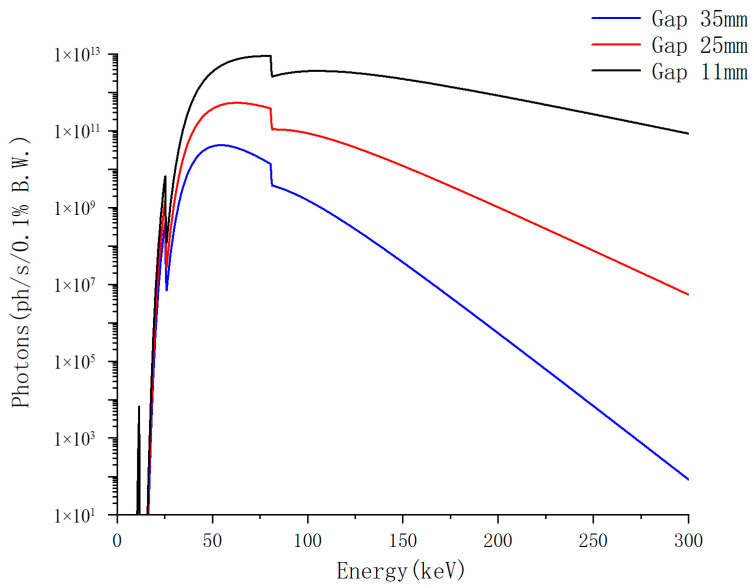
Calculated transmission spectra of the wiggler W73 source at the HEPS HXI beamline under different gap settings.

As shown in Figure 5, the transmission spectra were calculated for the wiggler W73 source at the HEPS HXI beamline with gap settings of 11 mm, 25 mm, and 35 mm, using a filter configuration of 0.1 mm Ag + 0.1 mm Au. The results clearly illustrate the dependence of the spectral distribution on the wiggler gap: a smaller gap yields a stronger magnetic field and higher deflection parameter (*K*), resulting in both increased photon flux and an extended upper limit of the photon energy spectrum.

Filtering: Metal foil filters can absorb low-energy photons. By choosing metals with specific K-edges with different thicknesses, the central energy and bandwidth of the white beam can be partially adjusted. Filters also suppress fluorescence and background while preventing detector overload caused by excessive photon flux.

**Figure 6:**
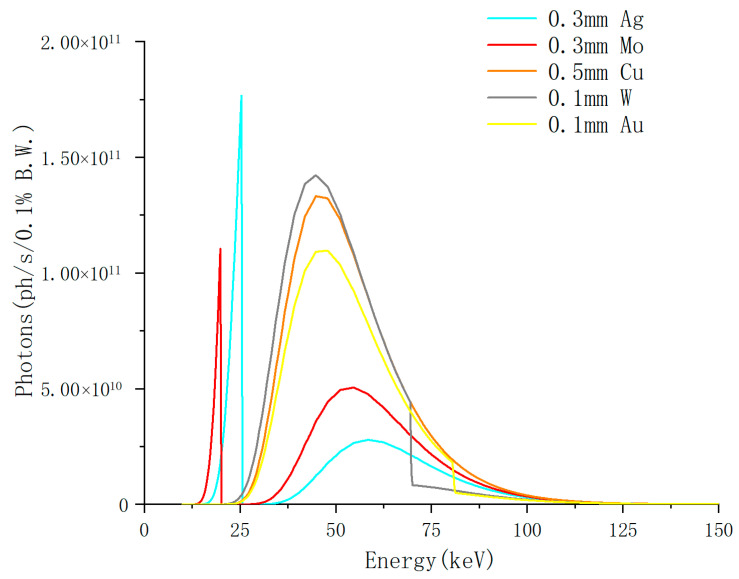
Calculated transmission of the wiggler W73 spectrum at the HEPS HXI beamline through different metal filters.

As shown in Figure 6, the transmission of the synchrotron spectrum was calculated for several common filters, based on the wiggler W73 source at the HEPS HXI beamline with a gap setting of 11 mm. The results revealed that filter material has a significant impact on spectral shaping: Ag or Mo filters retain more low-energy photons, whereas Cu, Au, or W filters preferentially transmit higher-energy photons.

Collimation and slits: Collimators and slits define the beam size and divergence, thereby controlling the gauge volume and the diffraction angle. The arrangement of the experimental geometry has been described in Section 3.1. In a typical setup, four slits are employed to maintain beam collimation and minimize divergence: two placed upstream of the sample and two between the sample and the detector, as shown in Figure 1. However, with fourth-generation synchrotron sources such as HEPS, the intrinsic beam divergence is extremely small (beam emittance 0.05~0.1 nm·rad) [44], which allows for a reduction in the number of upstream slits required before the sample.

### 6.2. Calibration and Corrections

Accurate quantitative EDXRD analysis requires correction for detector response, energy calibration, and systematic spectral effects. The quantum efficiency (QE) and energy resolution of detectors vary with photon energy, affecting the measured peak intensities and sensitivity of interplanar spacing d. Energy calibration is typically performed using well-known radioisotope sources, while geometrical calibration relies on diffraction from standards with known d-spacings at multiple energies to correct for instrumental deviations. Spectra must also be corrected for energy-dependent absorption in the sample and environment, Compton scattering producing a continuous background, and element-specific fluorescence that may overlap diffraction peaks. Regular calibration is essential for long-duration operando experiments to maintain quantitative accuracy.

### 6.3. Data Processing and Quantitative Methods

After calibration and correction, data processing begins with background subtraction, peak detection, and profile fitting, typically using Voigt or pseudo-Voigt functions. Overlapping peaks from multiple phases or diffraction orders require careful analysis, often guided by prior structural knowledge. Lattice parameters, phase information, and microstructural features can then be extracted based on peak statistics and resolution.

## 7. Representative Applications

### 7.1. High-Pressure Science

Robert Farla explored the high-energy wiggler beamline P61, demonstrating that it provides high-flux white beams for in situ studies at P61A and P61B, where the six-ram LVP Aster-15 enables ultra-high-pressure and high-temperature experiments with fast, high-quality EDXRD and radiography [24]. Kozaburo Tamura investigated fluid structures under high temperature and pressure using energy-dispersive X-ray scattering, highlighting improvements with high-energy X-ray tubes and modern synchrotron sources for better statistics and wider k-range data [46]. Yanbin Wang demonstrated the CAESAR technique for fast, efficient diffraction in limited-access setups, combining energy- and angle-dispersive methods [52]. Ho-Kwang Mao highlighted that energy-dispersive X-ray diffraction with polychromatic synchrotron microbeams enables high-resolution structural studies of microscopic samples in diamond-anvil cells at ultrahigh pressures and temperatures [53].

### 7.2. Battery Research

Amy C. Marschilok highlighted that EDXRD enables non-destructive studies of battery electrodes in their native housings, revealing system-level phenomena without altering cell construction [51]. Alyssa M. Stavola measured changes in battery composition and lattice parameters with high spatial resolution during cycling using in situ EDXRD at APS [87]. David J. Arnot investigated lithium battery electrodes, showing that operando spatially-resolved EDXRD and synchrotron tomography reveal how silver vanadium oxide (SVO) reduces reaction heterogeneity and how hybrid SVO/CFx cathodes optimize electron transfer, providing insights into current distribution, heat dissipation, and electrode composition [88].

### 7.3. Stress and Strain Studies

J.-S. Park demonstrated high-energy EDXRD at APS for non-destructively mapping residual strain in engineering and biological materials with mm-scale spatial resolution, multi-component detection, and complementary tomography, enabling precise analysis and in situ experiments [89]. M. Croft used high-energy synchrotron X-ray diffraction to map 2D strain fields in 4140 steel fatigue specimens, revealing how overload cycles create persistent compressive and tensile strain regions that influence crack growth retardation and in situ strain responses under varying loads [90]. He also studied elastic and plastic strain evolution in Ti–6Al–4V under four-point bending using synchrotron EDXRD, quantifying strain profiles, elastic modulus, plastic onset, and stress–strain behavior with high precision (≈1.5 × 10^−5^) [91].

### 7.4. Catalysis and Chemical Reactors

Ruggero Caminiti reviewed a new EDXRD method for the real-time monitoring of phase transition kinetics, offering simple measurements and straightforward data analysis, with applications to polymeric, biological, and mineralogical systems [92]. K. Ellmer described in situ EDXRD using synchrotron light to monitor nucleation and the growth of thin films during magnetron sputtering, demonstrated for tin-doped indium oxide (ITO) films [93]. Stefan Zander used in situ EDXRD and UV–Vis spectroscopy to study the aging of Cu/Zn hydroxide carbonate precursors, revealing how pH, temperature, and additives control zinc incorporation into malachite, which is key to Cu/ZnO catalyst synthesis [94].

## 8. Future Outlook

EDXRD is poised for significant development due to advances in detector technology, computational methods, and synchrotron X-ray source capabilities. From our perspective, several key directions will shape its future outlook:

### 8.1. Detector Development and Tradeoffs

The ideal EDXRD detector will combine HPGe-like energy resolution, CdTe-like high-energy stopping power, room-temperature operation, and high count-rate capability. Engineering compromises remain necessary to balance resolution, efficiency, and count-rate performance for different experiments.

### 8.2. Diffraction Source Advances

Development of EDXRD methodologies at synchrotron facilities is essential to fully exploit the unique advantages of these sources. Synchrotron radiation provides high photon flux, a broad energy spectrum, and rapid imaging capabilities, enabling high-throughput experiments, time-resolved studies, and investigations under extreme conditions. Optimized synchrotron EDXRD setups allow researchers to maximize the potential of the source, opening new opportunities for diffraction experiments.

### 8.3. Standardization and Automated Workflows

Establishing standardized and automated workflows is essential for improving the reproducibility and efficiency of EDXRD experiments. Standard protocols for energy calibration, detector efficiency reporting, and data correction ensure consistent and reliable results. Automated measurement and analysis reduce user-dependent variability and enable high-throughput data collection. The acquisition of large datasets, combined with open data sharing, lays the foundation for machine-learning-assisted diffraction.

### 8.4. Computational and Real-Time Analysis Advances

The integration of advanced data analytics and machine learning into EDXRD represents a promising frontier. Physical models linking EDXRD signals to diffusion, phase transitions, and mechanical behavior, combined with real-time data analysis, enable dynamic experiment guidance and more complicated experimental design. This enhances experimental efficiency and delivers deeper scientific insights.

## 9. Conclusions

EDXRD has become a versatile tool for studying structural changes in materials under realistic and extreme conditions. Synchrotron white beams, advanced HPGe detectors, and modern data processing now make previously impractical experiments feasible. Remaining challenges include improving detectors, standardizing procedures, and enabling laboratory portability. Continued advances will broaden EDXRD’s impact in materials science, energy storage, catalysis, and beyond.

## Data Availability

No new data were created or analyzed in this study. Data sharing is not applicable to this article.

## References

[1] Friedrich W., Knipping P., Laue M. (1913). Interferenzerscheinungen bei Röntgenstrahlen. Ann. Phys..

[2] Scherrer P. (1912). Bestimmung der inneren Struktur und der Größe von Kolloidteilchen mittels Röntgenstrahlen. Kolloidchemie: Ein Lehrbuch.

[3] Ewald P.P. (1962). Fifty Years of X-Ray Diffraction.

[4] Bragg W.H., Bragg W.L. (1913). Proceedings of the Royal Society of London. Series A, Containing Papers of a Mathematical and Physical Character.

[5] Birkholz M. (2005). Thin Film Analysis by X-Ray Scattering.

[6] Dinnebier R.E., Billinge S.J.L. (2008). Front matter. Powder Diffraction.

[7] Epp J. (2016). X-ray diffraction (XRD) techniques for materials characterization. Materials Characterization Using Nondestructive Evaluation (NDE) Methods.

[8] Khan H., Yerramilli A.S., D’Oliveira A., Alford T.L., Boffito D.C., Patience G.S. (2020). Experimental methods in chemical engineering: X-ray diffraction spectroscopy—XRD. Can. J. Chem. Eng..

[9] Spieß L., Behnken H., Genzel C., Schwarzer R., Teichert G. (2009). Moderne Röntgenbeugung: Röntgendiffraktometrie für Materialwissenschaftler, Physiker und Chemiker.

[10] Chauhan A. (2014). Powder XRD Technique and its Applications in Science and Technology. J. Anal. Bioanal. Tech..

[11] Quinn P.S., Benzonelli A., López Varela S.L. (2018). XRD and Materials Analysis. The Encyclopedia of Archaeological Sciences.

[12] Henke B.L., Gullikson E.M., Davis J.C. (1993). X-ray interactions: Photoabsorption, Scattering, Transmission, and Reflection at E = 50–30,000 eV, Z = 1–92. At. Data Nucl. Data Tables.

[13] Manova D., Mändl S. (2019). In situ XRD measurements to explore phase formation in the near-surface region. J. Appl. Phys..

[14] Fewster P.F. (2023). The limits of X-ray Diffraction Theory. Crystals.

[15] Jung D.Š., Lukin S., Halasz I. (2023). Improving the Accuracy of Small-Molecule Crystal Structures Solved from Powder X-ray Diffraction Data by Using External Sources. Helv. Chim. Acta.

[16] Buras B., Chwaszczewska J., Szarras S., Szmid Z. (1968). Fixed Angle Scattering (FAS) Method for X-ray Crystal Structure Analysis. Inst. Nucl. Res. Rep..

[17] Giessen B.C., Gordon G.E. (1968). X-ray Diffraction: New High-Speed Technique Based on X-ray Spectrography. Science.

[18] Laine E., Lähteenmäki I. (1980). The energy dispersive x-ray diffraction method: Annotated bibliography 1968–1978. J. Mater. Sci..

[19] Egami T., Güntherodt H.-J., Beck H. (1981). Structural Study by Energy dispersive X-Ray diffraction. Glassy Metals I.

[20] Kämpfe B., Luczak F., Michel B. (2005). Energy-Dispersive X-Ray Diffraction. Part. Part. Syst. Charact..

[21] Russ J.C. (1984). Fundamentals of Energy Dispersive X-Ray Analysis.

[22] APS Beamline 6-BM-A,B. APS Beamline Directory. Advanced Photon Source, U.S.. https://www.aps.anl.gov/Beamlines/Beamline-Directory/213.

[23] 27-ID HEX: High Energy Engineering X-Ray Scattering. NSLS-II Beamline Directory. Brookhaven National Laboratory 2026. https://www.bnl.gov/nsls2/beamlines/beamline.php?r=27-ID.

[24] Farla R., Bhat S., Sonntag S., Chanyshev A., Ma S., Ishii T., Liu Z., Néri A., Nishiyama N., Faria G.A. (2022). Extreme conditions research using the large-volume press at the P61B endstation, PETRA III. J. Synchrotron Rad..

[25] Drakopoulos M., Connolley T., Reinhard C., Atwood R., Magdysyuk O., Vo N., Hart M., Connor L., Humphreys B., Howell G. (2015). I12: The Joint Engineering, Environment and Processing (JEEP) beamline at Diamond Light Source. J. Synchrotron Rad..

[26] Pandey K.K., Poswal H.K., Mishra A.K., Dwivedi A., Vasanthi R., Garg N., Sharma S.M. (2013). Energy-dispersive X-ray diffraction beamline at Indus-2 synchrotron source. Pramana J. Phys..

[27] Pennicard D., Struth B., Hirsemann H., Sarajlic M., Smoljanin S., Zuvic M., Lampert M.O., Fritzsch T., Rothermund M., Graafsma H. (2014). A germanium hybrid pixel detector with 55 μm pixel size and 65,000 channels. J. Instrum..

[28] Förster A., Brandstetter S., Schulze-Briese C. (2019). Transforming X-ray detection with hybrid photon counting detectors. Phil. Trans. R. Soc. A.

[29] Planck M. (1901). Ueber das Gesetz der Energieverteilung im Normalspectrum. Ann. Phys..

[30] Gerhard K., Nicole M. (2009). Planck’s blackbody radiation law: Presentation in different domains and determination of the related dimensional constant. arXiv.

[31] Harrington G.F., Santiso J. (2021). Back-to-Basics tutorial: X-ray diffraction of thin films. J. Electroceram..

[32] Cullity B.D., Stock S.R. (2014). Elements of X-Ray Diffraction.

[33] Alford T.L., Feldman L.C., Mayer J.W. (2007). Fundamentals of Nanoscale Film Analysis.

[34] Strüder L., Lechner P., Leutenegger P. (1998). Silicon drift detector—The key to new experiments. Sci. Nat..

[35] Darken L.S., Cox C.E. (1995). High-Purity Germanium Detectors. Semiconductors and Semimetals.

[36] Li W., Guo S., Lai W., Li Z., Wang Z., Dai D. (2024). Calibration study of a silicon drift detector on a monoenergetic X-ray radiation device. IET Conf. Proc..

[37] Garrity D.J., Wenman M.R., Jenneson P.M., Courtney T.P., Vincent S.M. (2009). Development of a transmission geometry X-ray diffraction system for measuring the austenite to martensite phase transformation in 304 L stainless steel. Nucl. Instrum. Methods Phys. Res. A.

[38] Garrity D.J., Jenneson P.M., Crook R., Vincent S.M. (2010). Transmission diffraction-tomography system using a high-energy X-ray tube. Appl. Radiat. Isot..

[39] Abdelkader M.H., Alkhateeb S.M., Bradley D.A., Pani S. (2012). Development and characterization of a laboratory based X-ray diffraction imaging system for material and tissue characterization. Appl. Radiat. Isot..

[40] Sosa C., Malezan A., Poletti M.E., Perez R.D. (2017). Compact energy dispersive X-ray microdiffractometer for diagnosis of neoplastic tissues. Radiat. Phys. Chem..

[41] Vavrik D., Georgiev V., Jakubek J., Masek B., Urban O., Sleichrt J., Kytyr D. (2025). Transmission energy dispersive X-ray diffraction as a tool for the laboratory study of fast processes in metals. Sci. Rep..

[42] Helliwell J.R. (1998). Synchrotron radiation facilities. Acta Crystallogr. D Biol. Crystallogr..

[43] He J., Zhao Z. (2014). Shanghai synchrotron radiation facility. Natl. Sci. Rev..

[44] Xiaoming J., Jiuqing W., Qing Q., Yuhui D., Weifan S., Jian C., Gang X., Tiandou H., Hu D., Fusan C. (2014). The Chinese High-Energy Photon Source and its R&D Project. Synchrotron Radiat. News.

[45] Ellmer K., Mientus R., Wei V., Rossner H. (2003). In situ energy-dispersive x-ray diffraction system for time-resolved thin-film growth studies. Meas. Sci. Technol..

[46] Tamura K., Inui M., Hosokawa S. (1999). Energy-dispersive x-ray diffraction equipment for fluids at extreme conditions of high temperatures and high pressures. Rev. Sci. Instrum..

[47] Provis J.L., Van Deventer J.S.J. (2007). Geopolymerisation kinetics. 1. In situ energy-dispersive X-ray diffractometry. Chem. Eng. Sci..

[48] Chen Y., Wang X., Song Q., Xu J., Mu B. (2018). Development of a high-energy-resolution EDXRD system with a CdTe detector for security inspection. AIP Adv..

[49] O’Dwyer J.N., Tickner J.R. (2008). Quantitative mineral phase analysis of dry powders using energy-dispersive X-ray diffraction. Appl. Radiat. Isot..

[50] Chuang A.C., Park J.-S., Shade P.A., Schwalbach E.J., Groeber M.A., Musinski W.D. (2021). AFRL Additive Manufacturing Modeling Series: Challenge 1, Characterization of Residual Strain Distribution in Additively-Manufactured Metal Parts Using Energy-Dispersive Diffraction. Integr. Mater. Manuf. Innov..

[51] Marschilok A.C., Bruck A.M., Abraham A., Stackhouse C.A., Takeuchi K.J., Takeuchi E.S., Croft M., Gallaway J.W. (2020). Energy dispersive X-ray diffraction (EDXRD) for operando materials characterization within batteries. Phys. Chem. Chem. Phys..

[52] Wang Y., Uchida T., Von Dreele R., Rivers M.L., Nishiyama N., Funakoshi K., Nozawa A., Kaneko H. (2004). A new technique for angle-dispersive powder diffraction using an energy-dispersive setup and synchrotron radiation. J. Appl. Crystallogr..

[53] Mao H.-K., Hemley R.J. (1996). Energy dispersive x-ray diffraction of micro-crystals at ultrahigh pressures. High Press. Res..

[54] Ma Y., Mao H., Hemley R.J., Gramsch S.A., Shen G., Somayazulu M. (2001). Two-dimensional energy dispersive x-ray diffraction at high pressures and temperatures. Rev. Sci. Instrum..

[55] Genzel C.H., Denks I.A., Gibmeier J., Klaus M., Wagener G. (2007). The materials science synchrotron beamline EDDI for energy-dispersive diffraction analysis. Nucl. Instrum. Methods Phys. Res. A.

[56] Xhixha G., Alberi M., Baldoncini M., Bode K., Bylyku E., Cfarku F., Callegari I., Hasani F., Landsberger S., Mantovani F. (2016). Calibration of HPGe detectors using certified reference materials of natural origin. J. Radioanal. Nucl. Chem..

[57] Xhixha G., Bezzon G.P., Broggini C., Buso G.P., Caciolli A., Callegari I., De Bianchi S., Fiorentini G., Guastaldi E., Kaçeli Xhixha M. (2013). The worldwide NORM production and a fully automated gamma-ray spectrometer for their characterization. J. Radioanal. Nucl. Chem..

[58] Dewey S.C., Kearfott K.J. (2008). Calibration Drift in a Laboratory High Purity Germanium Detector Spectrometry System. Health Phys..

[59] Helmer R.G., Hardy J.C., Iacob V.E., Sanchez-Vega M., Neilson R.G., Nelson J. (2003). The use of Monte Carlo calculations in the determination of a Ge detector efficiency curve. Nucl. Instrum. Methods Phys. Res. A.

[60] Konstantinova M., Germanas D., Gudelis A., Plukis A. (2021). Efficiency calibration of high-purity germanium detector using Monte Carlo simulations including coincidence-summing corrections: Volume source case. Physics.

[61] Thompson A.C., Attwood D.T., Howells M.R., Kortright J.B., Robinson A.L., Underwood J.H., Kim K.-J., Kirz J., Lindau I., Pianetta P. (2009). X-Ray Data Booklet.

[62] Wang Z., Grosseau-Poussard J.-L., Geandier G., Panicaud B. (2021). Stress distribution in depth of NiCr + Cr_2_O_3_ systems using high-energy synchrotron X-rays in transmission mode. J. Alloys Compd..

[63] Wang Z., Grosseau-Poussard J.-L., Panicaud B., Geandier G., Renault P.-O., Goudeau P., Boudet N., Blanc N., Rakotovao F., Tao Z. (2020). Viscoplasticity and growth strain parameters identification by full modelling optimization during the high temperature oxidation of Ni28Cr modified by the reactive element yttria or zirconium. Comput. Mater. Sci..

[64] Faurie D., Geandier G., Renault P.-O., Le Bourhis E., Thiaudière D. (2013). Sin2 ψ analysis in thin films using 2D detectors: Non-linearity due to set-up, stress state and microstructure. Thin Solid Films.

[65] Koyama T., Senba Y., Yamazaki H., Takeuchi T., Tanaka M., Shimizu Y., Tsubota K., Matsuzaki Y., Kishimoto H., Miura T. (2022). Double-multilayer monochromators for high-energy and large-field X-ray imaging applications with intense pink beams at SPring-8 BL20B2. J. Synchrotron Rad..

[66] Owens A. (2012). Synchrotron light sources and radiation detector metrology. Nucl. Instrum. Methods Phys. Res. A.

[67] GLP Series Planar HPGe Low Energy Radiation Detectors. AMETEK ORTEC 2026. https://www.ortec-online.com/products/radiation-detectors/high-purity-germanium-hpge-radiation-detectors/hpge-radiation-detector-types-how-choose/glp-series-planar-hpge-low-energy-radiation-detectors.

[68] Yagi N. (2014). Synchrotron Radiation. Comprehensive Biomedical Physics.

[69] Tamura N., Padmore H.A., Patel J.R. (2005). High spatial resolution stress measurements using synchrotron based scanning X-ray microdiffraction with white or monochromatic beam. Mater. Sci. Eng. A.

[70] Pehl R.H., Goulding F.S., Landis D.A., Lenzlinger M. (1968). Accurate determination of the ionization energy in semiconductor detectors. Nucl. Instrum. Methods.

[71] Collins S.M., Shearman R., Ivanov P., Regan P.H. (2020). The impact of high-energy tailing in high-purity germanium gamma-ray spectrometry on the activity determination of 224Ra using the 241.0 keV emission. Appl. Radiat. Isot..

[72] Nurgalejev R., Pohuliai S., Sokolov A., Gostilo V., Vanpaemel J. (2021). Spectrometric performance of a HPGe semi-planar detector with large diameter. Nucl. Instrum. Methods Phys. Res. A.

[73] Hafızoğlu N. (2024). Efficiency and energy resolution of gamma spectrometry system with HPGe detector depending on variable source-to-detector distances. Eur. Phys. J. Plus.

[74] Zahn G.S., Genezini F.A. (2021). Efficiency stability of HPGe detectors under distinct count rates. Braz. J. Rad. Sci..

[75] Sangsingkeow P., Berry K.D., Dumas E.J., Raudorf T.W., Underwood T.A. (2003). Advances in germanium detector technology. Nucl. Instrum. Methods Phys. Res. A.

[76] Korchak O., Carna M., Havranek M., Marcisovsky M., Tomasek L., Vrba V. (2015). Properties of irradiated CdTe Detectors. Proceedings of the 23rd International Workshop on Vertex Detectors (Vertex2014).

[77] Mele F., Quercia J., Abbene L., Benassi G., Bettelli M., Buttacavoli A., Principato F., Zappettini A., Bertuccio G. (2023). Advances in High-Energy-Resolution CdZnTe Linear Array Pixel Detectors with Fast and Low Noise Readout Electronics. Sensors.

[78] Agostini G., Ambrosino F., Antonelli M., Aquilanti G., Bellutti P., Bertuccio G., Borghi G., Bosisio L., Campana R., Cautero G. (2025). Silicon drift detector monolithic arrays for X-ray spectroscopy. Front. Detect. Sci. Technol..

[79] Mele F., Gandola M., Bertuccio G. (2021). SIRIO: A High-Speed CMOS Charge-Sensitive Amplifier for High-Energy-Resolution X-γ ray spectroscopy with semiconductor detectors. IEEE Trans. Nucl. Sci..

[80] Iniewski K. (2024). CdTe and CdZnTe Materials: Material Properties and Applications.

[81] Gandola M., Grassi M., Mele F., Dedolli I., Malcovati P., Bertuccio G. (2022). The sparse readout RIGEL Application Specific Integrated Circuit for Pixel Silicon Drift Detectors in soft X-ray imaging space applications. Nucl. Instrum. Methods Phys. Res. A.

[82] Hafizh I., Carminati M., Fiorini C. (2020). First Prototype of 2 × 2 SCARLET: Readout ASIC for Bump-Bonded SDD Array for Large Event Throughput. Proceedings of the 2020 IEEE Nuclear Science Symposium and Medical Imaging Conference (NSS/MIC).

[83] Lopesa A.G., Bertelli L., Tauhata L. (2018). Performance of detectors used in measurements of radioactive material deposited in tissues from intakes by wounds. Braz. J. Rad. Sci..

[84] Owens A., Bavdaz M., Andersson H., Gagliardi T., Krumrey M., Nenonen S., Peacock A., Taylor I., Tröger L. (2002). The X-ray response of CdZnTe. Nucl. Instrum. Methods Phys. Res. A.

[85] Scheiber C. (2000). CdTe and CdZnTe detectors in nuclear medicine. Nucl. Instrum. Methods Phys. Res. A.

[86] Kincaid B.M. (1977). A short-period helical wiggler as an improved source of synchrotron radiation. J. Appl. Phys..

[87] Stavola A.M., Sun X., Guida D.P., Bruck A.M., Cao D., Okasinski J.S., Chuang A.C., Zhu H., Gallaway J.W. (2023). Lithiation Gradients and Tortuosity Factors in Thick NMC111-Argyrodite Solid-State Cathodes. ACS Energy Lett..

[88] Arnot D.J., Vila M.N., Hill R.C., Kingan A., Zhong Z., Vo N.T., Drakopoulos M., Takeuchi E.S., Marschilok A.C., Takeuchi K.J. (2025). Deciphering the Evolution of Current Distribution in Hybrid Silver Vanadium Oxide/Carbon Monofluoride Cathodes within Lithium Primary Batteries. ChemPhysChem.

[89] Park J.-S., Chuang A.C., Okasinski J., Chen H., Shade P., Turner T.J., Stock S., Almer J. (2022). A New Residual Strain Mapping Program Using Energy Dispersive X-Ray Diffraction at the Advanced Photon Source. Exp. Mech..

[90] Croft M., Shukla V., Jisrawi N.M., Zhong Z., Sadangi R.K., Holtz R.L., Pao P.S., Horvath K., Sadananda K., Ignatov A. (2009). Mapping and load response of overload strain fields: Synchrotron X-ray measurements. Int. J. Fatigue.

[91] Croft M., Shukla V., Akdoğan E.K., Jisrawi N., Zhong Z., Sadangi R., Ignatov A., Balarinni L., Horvath K., Tsakalakos T. (2009). In situ strain profiling of elastoplastic bending in Ti–6Al–4V alloy by synchrotron energy dispersive X-ray diffraction. J. Appl. Phys..

[92] Caminiti R., Albertini V.R. (1999). The kinetics of phase transitions observed by energy-dispersive x-ray diffraction. Int. Rev. Phys. Chem..

[93] Ellmer K., Mientus R., Weiß V., Rossner H. (2001). Setup for in situ X-ray diffraction studies of thin film growth by magnetron sputtering. Nucl. Instrum. Methods Phys. Res. A.

[94] Zander S., Seidlhofer B., Behrens M. (2012). In situ EDXRD study of the chemistry of aging of co-precipitated mixed cu,zn hydroxycarbonates–consequences for the preparation of Cu/ZnO catalysts. Dalton Trans..

